# Marktdesign für die Gasmangellage

**DOI:** 10.1007/s10273-022-3307-0

**Published:** 2022-11-30

**Authors:** Axel Ockenfels

**Affiliations:** Department of Economics, Universität zu Köln, Universitätsstraße 22a, 50923 Köln, Deutschland

**Keywords:** D47, H12, Q41

## Abstract

Bisher ist unklar, wer wie viel Gas zu welchem Preis in einer Mangellage in Deutschland bekommt, wenn der Regulierer rationieren muss. Dies droht die Anreize in und im Vorfeld einer Mangellage zu verwässern. In diesem Beitrag wird die potenziell hilfreiche Rolle eines Zertifikatsmarktes für die Allokation und Bepreisung in der Mangellage in den Blick genommen.

## Abstract (en)

So far, it is unclear who will get how much gas at what price in a gas shortage situation in Germany when the regulator has to ration. This threatens to dilute incentives in and in advance of a shortage. This paper looks at the potentially helpful role of a market for gas usage certificates for allocation and pricing in a shortage situation.

Nach welchen Regeln Marktmechanismen und Marktpreise in der Gasmangellage ersetzt werden, beeinflusst maßgeblich, ob und wie stark die Gasnachfrager und -anbieter Vorsorge für die Mangellage treffen – und beeinflusst damit auch die Wahrscheinlichkeit, ob diese überhaupt eintritt.[1] In diesem Beitrag wird ein ökonomischer Rahmen für die Mangellage vorgeschlagen. Kern der Idee ist ein Zertifikatsmarkt, der im weiteren Sinne an Märkte für Emissionsberechtigungen (Ellerman et al., 2016; Ockenfels 2009) sowie an dem Vorschlag von Schlecht et al. (2022) zu handelbaren Gasnutzungszertifikaten angelehnt ist. Während Schlecht et al. (2022) den Schwerpunkt auf eine Umverteilung der ökonomischen Renten im Gasmarkt vor einer etwaigen Rationierung legen, liegt der Fokus hier auf der potenziell hilfreichen Rolle eines Zertifikatsmarktes für die Allokation und Bepreisung speziell in der Mangellage.

Auf den ersten Blick sieht es womöglich so aus, dass ein solcher zusätzlicher Markt nicht nötig ist und etwa die Bundesnetzagentur keinen Einfluss auf die Marktergebnisse und insbesondere den Gaspreis in der Mangellage hat oder haben sollte, weil sie als „Bundeslastverteiler" nur entscheiden soll, welche Nachfrager abgeschaltet werden sollen.[2] Doch wenn Abschaltungen notwendig sind, um ein Gleichgewicht von Angebot und Nachfrage zu erzwingen, existiert definitionsgemäß kein Preis, der den Markt räumen könnte. Mit den Abschaltungen beeinflusst die Bundesnetzagentur also (die Erwartungen über) den Preis und über die gehandelten Mengen, zumindest implizit. Sie beeinflusst den Gaspreis direkt z. B. durch ihren durch die Abschaltungen ausgeübten Einfluss auf die Nachfrage. Dabei ist bisher wenig darüber bekannt, nach welchen Regeln die Abschaltungen erfolgen und wie diese genau von der Marktsituation abhängen. Gleichzeitig muss sie bei den Interventionen darauf achten, dass die Marktmacht der Gasanbieter bei extremer Knappheit diszipliniert wird. Für die abzuschaltenden Gasverbraucher müssen gegebenenfalls Kompensationen gezahlt werden. Für die nicht-rationierten Gasverbraucher, die nun möglicherweise von anderen Gasversorgern versorgt werden als ursprünglich vereinbart, muss festgelegt werden, welcher Preis zu entrichten ist. Zusätzlich kann es in der Mangellage auch zu politischen Einflussnahmen durch Preisbremsen und anderen Unterstützungsprogrammen kommen. All dies beeinflusst Preise und Preiserwartungen und sollte daher in einem expliziten und transparenten ökonomischen Rahmen geschehen, in dem sich diese effizient bilden können und Anreize schaffen, sich bestmöglich auf die Mangellage vorzubereiten.

## Ein neuer Zertifikatsmarkt

*Gasbasispreis*: Der Regulierer legt einen Gasbasispreis fest, der sich z. B. an den Preisen vor der Mangellage orientieren kann. Gerade auch für den Fall, dass in der Mangellage kein Marktpreis existiert und Abschaltungen notwendig werden – weil etwa die Nachfrage so unelastisch ist, dass kein Preis zu einem Ausgleich mit dem Angebot

**Prof. Dr. Axel Ockenfels** lehrt an der Fakultät für Wirtschafts- und Sozialwissenschaften und leitet das Center for Social and Economic Behavior (C-SEB) der Universität zu Köln.

1 Dasselbe gilt für Notfallszenarien in Strommärkten (Cramton et al., 2013) und bei Pandemien (Ockenfels, 2021), in denen rationiert wird. Siehe auch Cramton et al. (2020) sowie Ockenfels und Wambach (2022).

2 Existierende Regelenergieprodukte, das neue „Gasauktions-Modell" sowie der Einkauf von „Strategic Storage Based Options" sind hilfreiche marktliche Maßnahmen zur Bewältigung einer Gasmangellage, müssen allerdings durch weitere Maßnahmen ergänzt werden (Bundesnetzagentur, 2022).

* Dieser Beitrag profitierte von einem Austausch mit vielen Experten, besonders Peter Cramton, Sebastian Kranz, Christoph Maurer, Achim Wambach und Georg Zachmann. Ich bedanke mich für den wertvollen Input. Die Einschätzungen und Vorschläge spiegeln meine Ansicht wider. Ich bedanke mich auch beim European Research Council (GA No 741409 – EEC) und der Deutschen Forschungsgemeinschaft (EXC 2126/1– 390838866) für die Unterstützung meiner Forschung.

führen kann – kann (und muss) ein Gaspreis administrativ determiniert werden, implizit oder explizit.

In der Mangellage ist „trotz aller umgesetzten einschlägigen marktbasierten Maßnahmen“ das Angebot unzureichend (Bundesnetzagentur, o. D.; BMWi, 2019). Also ist das Gasangebot in der Mangellage zu jedem Zeitpunkt ausgereizt, d. h. unelastisch. Nur eine Nachfragereduktion kann dann zu einem Ausgleich von Angebot und Nachfrage führen. Der Nachfrageüberhang wird durch den Zertifikatspreis und gegebenenfalls weiteren Entscheidungen im Zertifikatsmarkt beseitigt.

*Zertifikatspreis*: Alle Nachfrager, die Gas verbrauchen, müssen nicht nur den Gasbasispreis zahlen, sondern auch ein Zertifikat über die verbrauchte Menge vorlegen, d.h. eine Berechtigung zum Gaskonsum. Der Regulierer beschränkt die Menge dieser Zertifikate auf das in der Mangellage jeweils verfügbare Gas. Die Zertifikate können in einer vom Regulierer organisierten Auktion oder in Sekundärmärkten erworben werden, oder werden kostenlos zugeteilt. Analog zum europäischen $CO_2$-Emissionshandel führt der Zertifikatshandel zu einer Zuteilung der Zertifikate nach Zahlungsbereitschaften und einem Zertifikatspreis, der die Knappheit reflektiert.

In der Mangellage gibt es Gründe, das knappe Gas nicht allein nach Zahlungsbereitschaften zu verteilen. Ein Vorteil des Zertifikatsmarktes ist es, dass durch die Ausgabe verschiedener Varianten von Zertifikaten die politischen und regulatorischen Präferenzen bei der Zuteilung abgebildet werden können. Dies geschieht durch den Einsatz von drei Zertifikatsvarianten:

1. *Standard-Zertifikate* werden versteigert. Jeder Nachfrager kann die Zertifikate zum Auktionspreis oder im Sekundärmarkt zum Marktpreis kaufen. Der Anreiz zum Gassparen ist proportional zur Summe von Gasbasispreis und Zertifikatspreis.

2. *Frei zugeteilte handelbare Zertifikate* werden privilegierten und geschützten Nachfragern kostenlos zugeteilt. Beispielsweise könnten Unternehmen und Haushalte Zertifikate erhalten, die 80 % ihres Vorjahresverbrauchs decken. Ein etwaiger Mehrbedarf muss hinzugekauft werden, Minderbedarfe führen zu Zertifikatsverkäufen. Dadurch können ausgesuchte Gasnachfrager entlastet werden, die aus sozialen oder ökonomischen Gründen nicht in der Lage sind oder denen man nicht zumuten möchte, dass sie (alle) benötigten Zertifikate am Markt erwerben. Gleichzeitig gilt, dass auch hier der Anreiz zum Gassparen proportional zur Summe von Gasbasispreis und Zertifikatpreis ist, da der Zertifikatspreis auch bei freier Zuteilung Opportunitätskosten darstellt.

3. *Frei zugeteilte nicht-handelbare Zertifikate* werden ebenso privilegierten und geschützten Gasnachfragern kostenlos zugeteilt. Allerdings gilt hier die Auflage, dass das den zugeteilten Zertifikaten entsprechende Gas auch tatsächlich verbraucht werden muss und jedenfalls nicht auf dem Markt veräußert werden darf. Daher ist bei diesen Nachfragern der Anreiz zum Gassparen lediglich proportional zum Gasbasispreis und damit geringer als bei den anderen Zertifikatsvarianten.

So könnte Krankenhäusern untersagt werden, Gasgeschäfte gegen die Gewinne durch die Krankenversorgung abzuwägen (Zertifikatsvariante 3), während dem Bäcker eine Belohnung winkt, wenn er seinen Gasverbrauch reduziert und dadurch Zertifikate gewinnbringend veräußern kann (Zertifikatsvariante 2). Für Haushalte kann der Regulierer die benötigte Menge nicht-handelbarer Zertifikate pauschal vorhalten und entsprechend vom Zertifikatsmarkt fernhalten. Alternativ können Haushalte Anreize erhalten, gemäß Zertifikatspreis für Gaseinsparungen entlohnt zu werden. So kann der Zertifikatsmarkt Prämien für Gaseinsparungen generieren. Wenn die Zertifikate in den Varianten 2 und 3 gemäß den politischen und regulatorischen Präferenzen und Notwendigkeiten vergeben sind, kann der Zertifikatshandel dafür sorgen, dass Markt- und Preismechanismen ihren größtmöglichen Beitrag zur Lösung des Allokationsproblems in der Mangellage leisten können.

*Marktergebnis*: Im Ergebnis ist der Nachfragepreis für Gas gleich der Summe von festgelegtem Gasbasispreis und endogenem Zertifikatspreis, nachdem die nicht-handelbaren Zertifikate (3) für privilegierte und geschützte Kunden vorab bedient wurden. Zugleich zeigt der Nachfragepreis die Opportunitätskosten einer bevorzugten Zuteilung an privilegierte und geschützte Nachfragern an. Im Gleichgewicht wird das verfügbare Gas in der Mangellage vollständig und marktgerecht unter den politischen und angebotsseitigen Nebenbedingungen verteilt.

Die durch die hohe Nachfrage entstehenden Renten des Zertifikatsmarkts gehen nicht an die Gasanbieter, sondern an den Staat bzw. an die Empfänger frei zugeteilter Zertifikate. Diese an den Staat gehenden Renten können eingesetzt werden, um das Gasangebot zu finanzieren bzw. den Gasbasispreis zu reduzieren. Rationierten Nachfragern wird untersagt, Zertifikate zu kaufen.

Der Zertifikatsmarkt erlaubt dem Regulierer eine „faire“ Lastenverteilung vorzunehmen und die Rationierung in einem einheitlichen Rahmen umzusetzen. Er kann dem Regulierer aber nicht die Entscheidung abnehmen, welche Nachfrager nicht-handelbare Zertifikate erhalten oder rationiert werden, oder inwieweit bestimmte Nachfrager entlastet werden sollen. Das ist die Aufgabe der Politik und Regulierung.

### Vorteile des Zertifikatsmarkts

*Erstens* ermöglicht er dem Regulierer, durch die Zuteilung von unterschiedlichen Zertifikatsvarianten präzise, transparent und flexibel zu entscheiden, wie viel Markt ermöglicht wird, und welchen Nachfragern eine Sonderbehandlung bei der Zuteilung und den Kosten zugutekommt, und unter welchen Bedingungen (handelbare versus nicht handelbare Zertifikate).

*Zweitens* kommt unter solchen politischen und regulatorischen Nebenbedingungen den Preissignalen und Opportunitätskosten des Gasverbrauchs die maximal mögliche Rolle bei der Zuteilung zu, und zwar selbst dann, wenn der Gasmarkt in der Mangellage ansonsten nicht mehr funktionsfähig ist und wenn ein Großteil der Zertifikate kostenlos zugeteilt würde.

Dies gilt *drittens* besonders auch für die Unternehmen und Nachfrager, deren Preissensitivität ohne das Erfordernis, ein Zertifikat vorzulegen, andernfalls sehr gering wäre. Dies könnte etwa bei Unternehmen der Fall sein, die den Preis schon länger abgesichert haben, oder bei Nachfragern mit nur begrenzt anpassbaren Verträgen.

Und *viertens* sichert die Nachfragereduktion über den Zertifikatsmarkt statt über den Gasmarkt, dass die Kosten des Überbietungswettbewerbs der Nachfrage, die über die Angebotskosten hinausgehen, nicht an die Gasanbieter gehen, sondern der Entlastung der Nachfrage dienen können.

### Herausforderungen

Der Terminmarkt ist nicht inkompatibel mit einer (zusätzlichen) Bepreisung einer physischen Gaslieferung. Allerdings können Terminkontrakte bei einer Rationierung nicht oder nicht alle bedient werden, sodass ein anderes Verfahren der Gasallokation gefunden werden muss. Der Zertifikatsmarkt ist ein solches Verfahren. Es gibt jedoch viele Herausforderungen, die vor einer Implementierung eines Zertifikatsmarktes gelöst werden müssen. Eine Frage ist der Umgang mit der Unsicherheit darüber, wie sich Angebot und Nachfrage in der Mangellage entwickeln. Diese entsteht z.B., da Haushalte nicht am Handel teilnehmen, sodass der Haushaltsverbrauch relativ unkontrolliert bleibt. Eine Möglichkeit ist, die Mangellage weiter in Unterstufen zu unterteilen, sodass eine höhere Stufe mit zunehmender Rationierung (also weniger Zertifikatsangebot) verbunden wird. Alternativ kann das Zertifikatsangebot als Basiswert multipliziert mit Krisenindex definiert werden, wobei der Krisenindex je nach Knappheitssituation über die Zeit variieren kann, um die Gesamtgasnutzung über die Zeit zu steuern. Die Gültigkeit der Zertifikate und inwieweit sie angesichts der Speicherbarkeit von Gas aufgespart werden können – zum Speicherpreis – muss festgelegt werden. Ohne eine internationale Koordination des Zertifikatsmarktes können Ineffizienzen resultieren. Dies gilt für alle nationalen Zuteilungsansätze. Schließlich ist es eine Herausforderung, einen Zertifikatsmarkt mitsamt seinen Anforderungen an Monitoring, Compliance und Enforcement noch für den kommenden Winter rechtssicher umzusetzen. Doch scheinen hier die Herausforderungen nicht größer als bei anderen Ansätzen, die sich dem Zuteilungsproblem in der Mangellage systematisch annehmen und dem Markt trotz Rationierung einen möglichst großen Raum geben wollen. Umgekehrt darf die Vorbereitung eines ökonomischen Rahmens nicht dazu verleiten, zu früh in die Rationierung überzugehen, oder zu lange darin zu verharren, um zusätzliche Entlastungen etwa für Teile der heimischen Industrie zu erreichen. Unabhängig davon, wie genau die Regeln für die Allokation und Preise des verfügbaren Gases in der Mangellage letztlich ausgestaltet werden: Je früher dies geschieht, desto stärker können die damit verbundenen Anreize bereits im Vorfeld und inmitten der Mangellage wirken.

**Title:** ***Market Design for a Gas Shortage***
